# Early breast cancer detection and differentiation tool based on tissue impedance characteristics and machine learning

**DOI:** 10.3389/frai.2023.1248977

**Published:** 2023-09-13

**Authors:** Soumaya Ben Salem, Samar Zahra Ali, Anyik John Leo, Zied Lachiri, Martin Mkandawire

**Affiliations:** ^1^SITI Laboratory, National School of Engineers of Tunis, University of Tunis El Manar, Tunis, Tunisia; ^2^Department of Chemistry, School Science and Technology, Cape Breton University, Sydney, NS, Canada

**Keywords:** malignant lesion, machine learning, artificial intelligence, mammography imaging, electrical impedance spectroscopy, cancer differentiation

## Abstract

During Basic screening, it is challenging, if not impossible to detect breast cancer especially in the earliest stage of tumor development. However, measuring the electrical impedance of biological tissue can detect abnormalities even before being palpable. Thus, we used impedance characteristics data of various breast tissue to develop a breast cancer screening tool guided and augmented by a deep learning (DL). A DL algorithm was trained to ideally classify six classes of breast cancer based on electrical impedance characteristics data of the breast tissue. The tool correctly predicted breast cancer in data of patients whose breast tissue impedance was reported to have been measured when other methods detected no anomaly in the tissue. Furthermore, a DL-based approach using Long Short-Term Memory (LSTM) effectively classified breast tissue with an accuracy of 96.67%. Thus, the DL algorithm and method we developed accurately augmented breast tissue classification using electrical impedance and enhanced the ability to detect and differentiate cancerous tissue in very early stages. However, more data and pre-clinical is required to improve the accuracy of this early breast cancer detection and differentiation tool.

## 1. Introduction

Breast cancer is the second leading cause of mortality worldwide in women (and rarely in men) after skin cancer. Unfortunately, new cases of breast cancer have been increasing for the last few decades. For instance, From 2008 to 2017, the number of new cases increased by half a year, reaching 11.7% in 2020 (Sung et al., 2021; American Cancer Society., 2022). As a result, over 2.3 million women were diagnosed with breast cancer, and 685 000 died of the disease globally in 2020. However, when diagnosed and treated during the early stages, breast cancer has approximately a 90% survival rate (Kubicek et al., 1970; Heywang-Köbrunner et al., 2011; Helwan et al., 2017).

Consequently, screening for early detection of breast tumors has been crucial in breast cancer interventions and treatment. There is a correlation between tumor size, usually related to early detection, and breast cancer deaths, decreasing the mortality rate by 1.3% per millimeter size decrease (Sanchez et al., 2013). Hence, early detection of breast tumors during basic screening is critical for achieving a good therapy for cancer, even before being palpable. Generally, X-Ray Mammography Imaging is the primary detection tool for breast cancer, widely used in breast screening for decades, especially in asymptomatic women (Malich et al., 2000). Although mammography imaging has led to a significant reduction in breast cancer deaths, the technique is sometimes inadequate for assessing, especially for women presenting symptoms that need complementary methods, such as additional imaging like Magnetic Resonance Imaging (MRI), Endoscopy, whole-breast ultrasound percutaneous, and breast biopsy may follow to confirm the findings (Jossinet, 1996, 1998; Al Amin et al., 2014; Zubair et al., 2021).

The potential for misdiagnosing cancer due to poor evaluation of abnormalities using imaging techniques is very common. For instance, X-Ray mammography imaging is less efficient in sensitivity and specificity (Perner and Imiya, 2011), notably in women under 50 years of age (Budak et al., 2019; Cai et al., 2019). Furthermore, the output images may contain many artifacts and noise, making it difficult to identify small lumps, especially in dense tissue. Invasive techniques, like biopsies, have also been attributed to the acceleration of the invasiveness of tumor growth (Estrela da Silva et al., 2000). Most of these techniques use high energy during the screening procedure to provide high-quality images for discrimination of abnormalities, which can be very painful to the patient and harm the human body, resulting in most women shying away, for example, from frequent or routine X-Ray mammography screening. Thus, the need for a screening technique that fulfills the demands of rapidity, earlier detection, lesion differentiation and non-invasiveness is still the major intention of researchers to reduce the breast cancer mortality rate (Kumar et al., 2020).

There has been a significant advancement in using electrical impedance in classifying various tissue types on a different but related front. Thus, it is possible to differentiate between healthy and cancerous tissue depending on electrical impedance behavior, even before the tumor becomes apparent. Electrical impedance has been used in Electrical Impedance Spectroscopy (EIS), an emerging technique for diagnosing abnormalities and tumor detection, and applied in several fields, including the classification of heart disease (Alvi et al., 2021), the study of biological tissue (Hope and Iles, 2003; Ng et al., 2008; Shorten and Khoshgoftaar, 2019), identification of low quantities of breast cancer cells (Estrela da Silva et al., 2000), blood volume changes during the heart cycle and characterization of human lung tissue (Estrela da Silva et al., 2000; Sanchez et al., 2013; Magar et al., 2021). Generally, cancerous tissue displays specific dielectric properties compared to benign tumors, identified by higher capacity (i.e., storage of electrical potential) and conductivity (i.e., the reciprocal of resistance of an alternating current) values (Cheng and Fu, 2018; Hussein et al., 2019).

Similarly, the electric impedance technique is gaining prominence in breast screening, providing early detection of tumors and discrimination of malignant from benign lesions. The breast tissue can be categorized into six classes using electric impedance measurements (Estrela da Silva et al., 2000). This classification provides basic information about the healthy mammary structure that helps distinguish between malignant, premalignant and benign lesions. Substantial data on the electrical impedance of breast tissue from patients have accumulated in a few databases, capable of differentiating between carcinoma and begin lesions with an accuracy of approximately 92% overall in the tissue classes (Jossinet, 1996, 1998; Estrela da Silva et al., 2000; Hope and Iles, 2003; Ng et al., 2008; Islam, 2013). Furthermore, using the high-resolution mode of EIS, malignant lesions are correctly classified with an accuracy of 93.1% and 65.5% for benign lesions (Malich et al., 2000; Moqadam et al., 2018).

Therefore, the present paper reports our research to develop an Artificial Intelligence (AI) system for early and non-invasive breast cancer detection using data from electrical impedance measures. AI is revolutionizing digital health care by using mathematical algorithms to imitate the human brain to make decisions and resolve problems. In general, an AI system works by ingesting large amounts of labeled training data, analyzing the data for correlations and patterns, and using these patterns to make predictions, or in the case of diseases, autonomously diagnosing diseases, especially cancer (van der Maaten and Hinton, 2008; Powers, 2011; Budak et al., 2019).

Within AI, machine learning (ML) allows systems to learn and improve from experience without being explicitly programmed automatically. In machine learning, there is deep learning that imitates the way humans gain certain types of knowledge. While traditional machine learning algorithms are linear, deep learning (DL) algorithms are stacked in increasing complexity and abstraction hierarchy. Our research adopted deep learning to develop a breast cancer differentiation, classification, and detection system based on breast tissue impedance measurement data.

Biological tissue can conduct electrical current with the association of impedance parameters. To better understand these electrical properties, it is important to consider some basics of tissue composition. Biological tissue comprises extracellular medium and cells; the latter consists of the cell membrane and intracellular medium composed of ionic solution and the extracellular medium. These ions in the solution are responsible for the electrical conductivity of cells (Malich et al., 2000; Hussein et al., 2019). A cell membrane acts dependently over the frequency in a simple RC circuit. Lower frequencies allow the cellular membrane to act like an insulator while the impedance is more resistive. At a higher frequency, the ability of the cell membrane to pass an electrical current is more important while the impedance decreases. Thus, biological tissues react dependently to the frequency after applying electrical current. Therefore, choosing the frequency range is important, especially for tumor detection.

## 2. Methods

### 2.1. Artificial intelligence: the implored techniques

AI is a computer system that imitates human reasoning ability to learn and solve issues. Due to AI techniques, the need for human intervention will become progressively less important, accompanied by the independent learning process that allows self-correction/adaption to enhance the performance of the system continuously. AI also has broad applications in the healthcare sector (Kumar et al., 2020); this technology can outperform human physicians by helping to prevent and treat diseases or disabilities, offer improved clinical decisions, provide rapid and early detection of ailments, diagnose illnesses including cancer, and hence predict health status (Kumar et al., 2020). The AI learning concept in digital healthcare has to fulfill the requirement of using large electronic health records to enable the system to follow characteristics and patterns detected by the ML virtual components.

**Figure 1:**
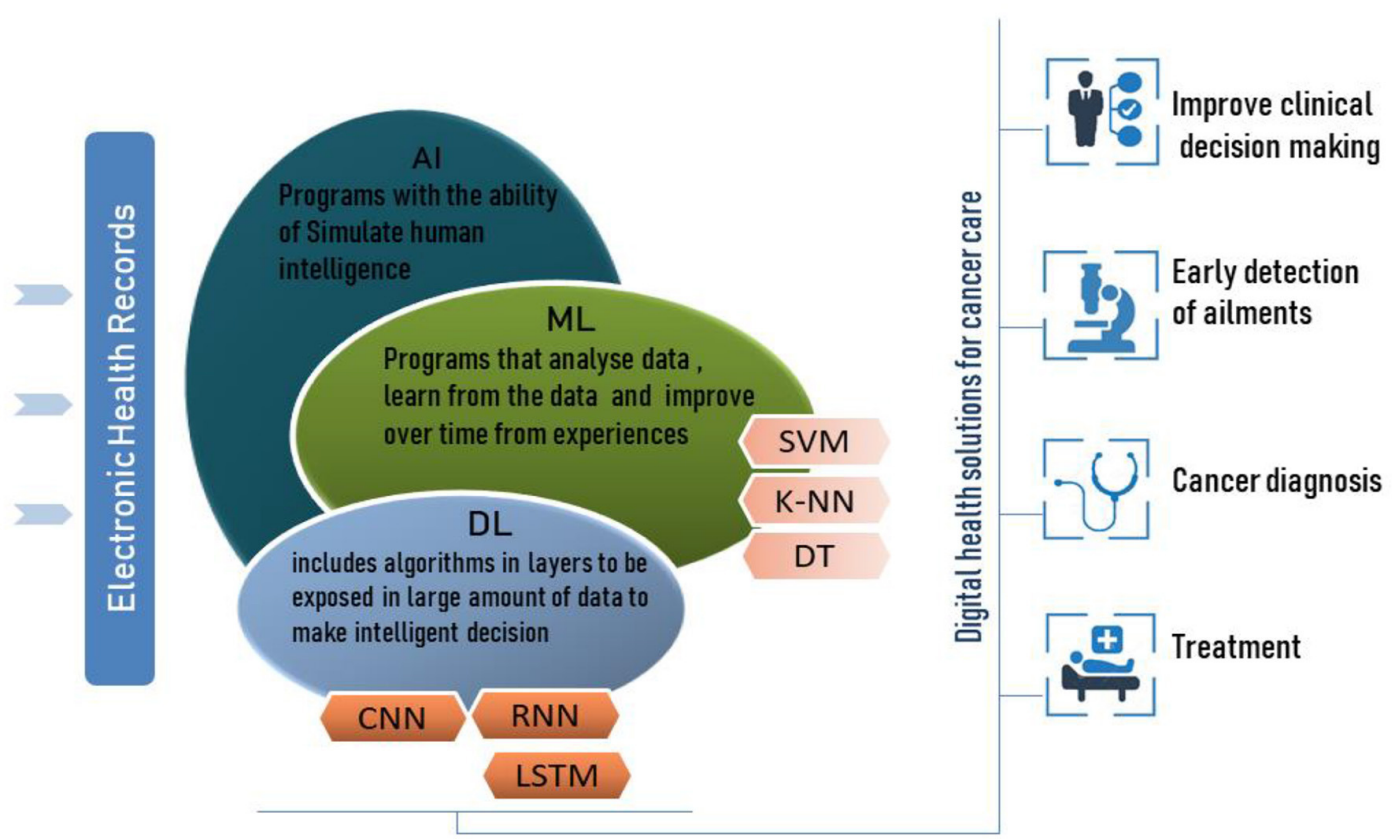
Relationships between AI, ML and DL for digital health solutions for cancer care.

Machine learning is a subfield of AI (Figure 1) that develops algorithms to analyze historical data, learn from this data and predict status to make informed decisions without explicit programming. ML can self-update its algorithm's parameters and change its internal programming in each iteration to better perform certain tasks. ML has enabled data-driven decisions or predictions of accurate future outcomes using various ML techniques such as Support Vector Machine (SVM) and K-nearest neighbor (K-NN).

Support Vector Machine (SVM) is a supervised machine learning technique for classification and regression issues. The SVM algorithm needs to find a hyperplane to separate classes through its support vectors with a maximized margin between each class.

SVM is considered a robust prediction method due to its ability to perform linear and non-linear classification with its kernel function that allows it to map inputs into higher dimensional feature space. Furthermore, the SVM algorithm is formulated to classify binary categories. This machine learning technique is adopted in this study to classify six different types of breast tissue, which requires constructing a multiclass error-correcting output codes (ECOC) model that combines multiple SVM binary classifiers.

Deep learning is considered an evolutional subfield of machine learning, and its algorithm is an intersection of advanced mathematics, statistics, and computer science to learn from the data and generate patterns to make informed decisions and predict results. Unlike machine learning, deep learning includes algorithms in layers to build an artificial neural network that can change its parameters during learning steps and compute features in each layer to predict intelligent decisions.

Long-Short-Term Memory (LSTM) is a special type of RNN suited to model a series of data points saved in time sequence due to its robust structure composed of recurrent connections and special memory blocks (Shorten and Khoshgoftaar, 2019). LSTM structure is composed of memory cells and self-connections; each cell state gets the flow of information from the input gate.

**Figure 2:**
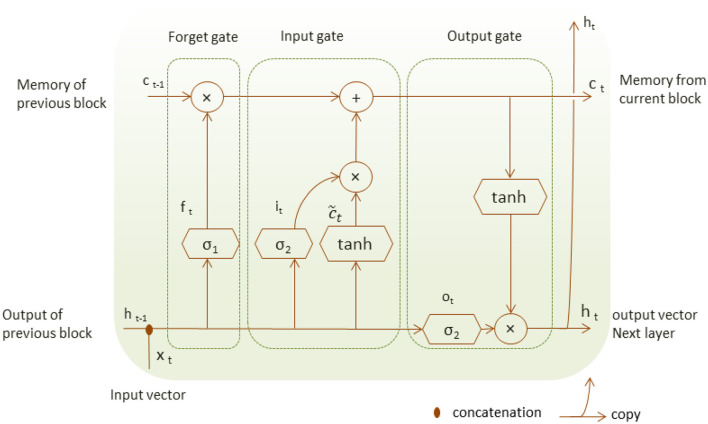
The LSTM unit structure.

Unlike RNN, LSTM structure adds a second hidden state or cell state and the concept of gates that control a memory cell activation vector (Hope and Iles, 2003). The input gate decides which part of the input x_t_ is saved to the cell state c_t_, the forget gate can forget or remember part of the accumulated memory of the cell state c_t−1_ at the previous time, and the current cell state c_t_, the output gate decides which information should be available at the current output value h_t_ of LSTM unit as shown in Figure 2. LSTM unit has four neural network layers of the forget gate, the input gate and the output gate that perform with the following operations.


(1) $$\begin{array}{l} {\text{f}}_{\text{t}}\;=\sigma_{1}\left({\text{w}}_{\text{f}}\left[{\text{h}}_{\text{t}-1},{\text{x}}_{\text{t}}\right]+{\text{b}}_{\text{f}}\right) \end{array}$$

(2) $$\begin{array}{l} {\text{i}}_{\text{t}}\;=\sigma_{2}\left({\text{w}}_{\text{i}}\left[{\text{h}}_{\text{t}-1},{\text{x}}_{\text{t}}\right]+{\text{b}}_{\text{i}}\right) \end{array}$$

(3) $$\begin{array}{l} {\text{o}}_{\text{t}}\;=\sigma_{3}\left({\text{w}}_{\text{o}}\left[{\text{h}}_{\text{t}-1},{\text{x}}_{\text{t}}\right]+{\text{b}}_{\text{o}}\right) \end{array}$$

The cell state and the cell output are defined respectively as follows:


(4) $$\begin{array}{l} {\text{c}}_{\text{t}}\;=f_{t}*c_{t-1\;}+\underset{{\tilde{c}}_{t}}{\underset{︸}{i_{t}*\text{tanh}\left({\text{w}}_{c}\left[h_{t-1},x_{t}\right]+b_{c}\right)}} \end{array}$$

(5) $$\begin{array}{l} {\text{h}}_{\text{t}}={\text{c}}_{\text{t}}*\text{tanh} \end{array}$$

Where σ and tanh are the neural network layers, they represent the neural sigmoid and hyperbolic tangent functions, respectively. The weight w and bias b represent the matrix of each of the three gates.

### 2.2. Data source

This study used a data set deduced from the application of electrical impedance spectroscopy on 64 patients undergoing breast surgery; measurements are taken in the first 30 min after the excision of breast tissue samples during the surgery. **Figure 4A** represents the different steps of data collection. The database includes six normal and pathological tissue classes, as shown in Table 1.

**Table 1:** The different breast tissue classes.

**Tissue classes**	**Spectra (cases)**	**Categories**	**Characteristics**
Carcinoma	21	Pathological tissue	Cancerous tissue
Fibro-adenoma	15		Benign tumor
Mastopathy	18		Benign disease (lumpy) Fibrocystic breast changes
Glandular	16	Normal tissue	Part of the breast that makes milk
Adipose	14		Fatty tissue
Connective	22		

During the data collection procedure, 12 impedance measurements from each EIS plot are computed in a frequency range from 488Hz to 1 MHz. which represent the alpha and beta frequency range (f < 1000Hz) and (1000Hz < f < 100 MHz). This particular region is useful for detecting tumors, according to some researchers.

### 2.3. Development of AI system

In order to detect the cell changes in real-time, an electrical current is applied to the aforementioned exciting tissues to create a spectrum, which is made by measuring the impedance parameters at different frequencies. One hundred twenty impedance spectra were recorded from each sample, and 14 spectra were discarded due to their erroneous features.

Due to the increasing interest in identifying the tissue's pathological properties, the complex impedance characteristics are investigated to interpret the change in the functional properties of the cell. Hence, Jossinet (1996, 1998) and Estrela da Silva et al. (2000) have been chosen to extract complex impedance features using the Agrand plane that helps to calculate the imaginary impedance part and the real impedance component of the plots. Table 2 shows the nine features extracted from the electrical impedance spectrum. The extracted features are of the size (106 × 9) and are used as the input data for both adopted methods (**Figure 4A**).

**Table 2:** The EIS features extracted for use in the machine learning.

**Symbol**	**Features**
I0	Impedivity (ohm) at zero frequency
PA500	Phase angle at 500 kHz
HFS	The high-frequency slope of the phase angle
DA	Impedance distance between spectral ends
AREA	Area Under Spectrum
AD/A	Area normalized by DA
IPmax	Maximum of the Spectrum
DR	Distance between I0 and the real part of the maximum frequency point
P	Length of the spectral curve

The entire data was divided randomly into training and testing separate subsets. In particular, the training set, representing about 80% of the whole dataset, is used to build the predictive model by taking advantage of the cross-validation (CV) approach. During the training procedure, the data is introduced to a multiclass ECOC model after creating the SVM template that requires the application of one of two filter types to transform the data and the use of the chosen kernel function among polynomial, linear or Radial Basic Function (RBF) which provides the window to project the data into a higher number of dimension spaces (**Figure 4B**).

The internal parameters of the model can highly affect the model's performance; therefore, to tune the model correctly, it is recommended to adjust its internal parameters with an optimization step that selects the values of the hyperparameters automatically. Different combinations of hyperparameter values are set in order to minimize the classification loss with the help of an optimization scheme.

**Figure 3:**
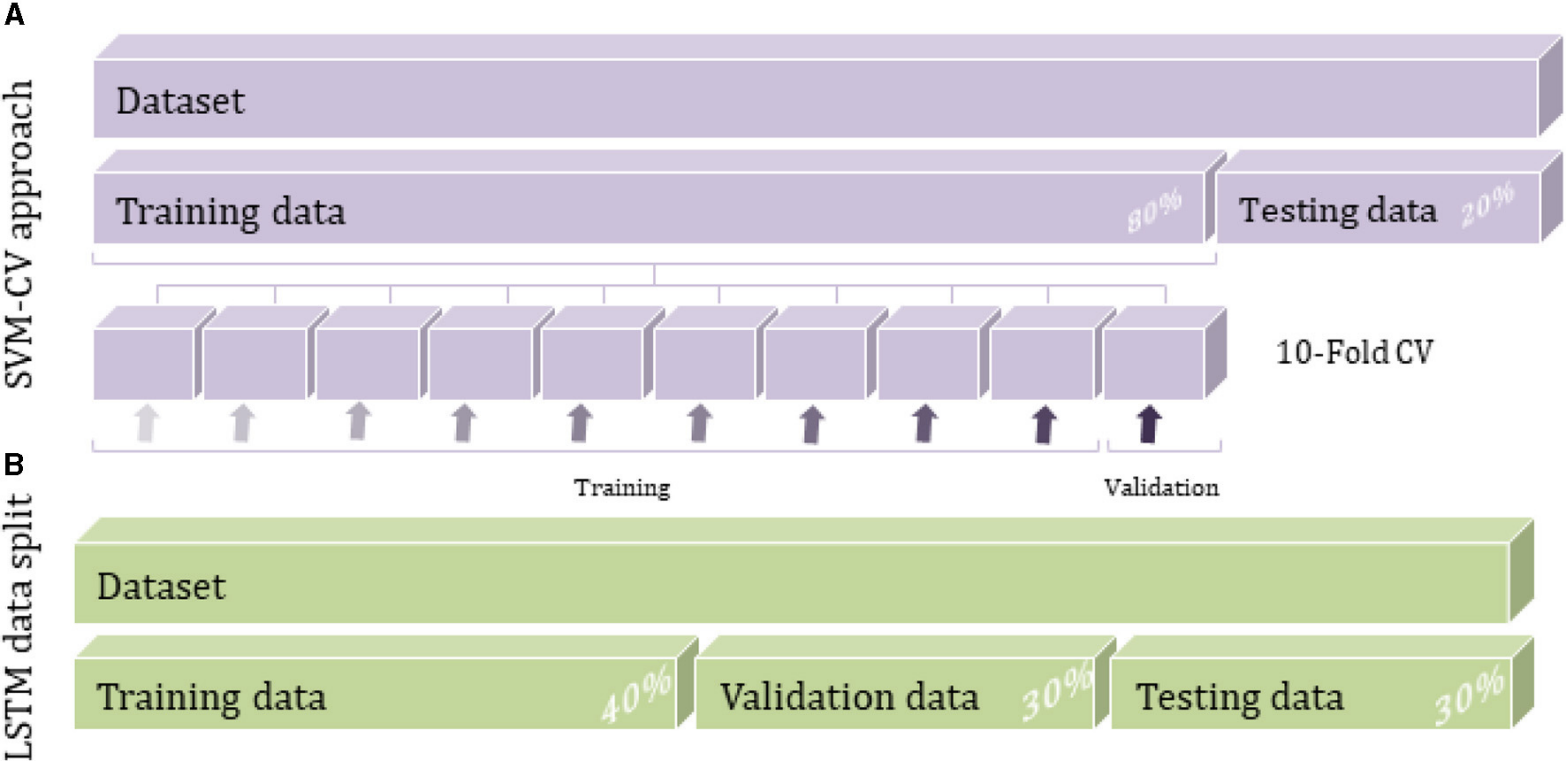
Data splitting techniques for SVM and LSTM methods: **(A)** SVM cross-validation approach and **(B)** LSTM data split method.

The CV approach is implemented during the model construction to estimate how accurately the model will perform. The CV algorithm randomly generates k partitions (folds) through the training data; each has an index number of 1 to k. The predictive model would run k iterations by taking interchangeably one fold as the validation set and the remaining k-1 folds as a training set until sweeping all the original data, as shown in Figure 3A. The predictive results are averaged over the k iterations to estimate the model's performance. Instantly, the hyperparameters optimization process seeks to reduce CV loss and improve classification accuracy.

A growing interest was observed over the last couple of years in a deep neural network for tabular data classification due to the great success achieved by their applications. As the dataset used in this study is static tabular data usually associated with ML techniques like K-NN and SVM (Alvi et al., 2021), its application in DL is extremely important to consider increasing the data size to train and evaluate deep learning algorithms effectively. The current dataset is relatively small to be used with the DL algorithm, so the data augmentation technique needs to be applied to the raw input data as a pre-processing step. The amount and diversity of the available data during training largely rely on the model's prediction accuracy. In particular, data augmentation approaches can prevent overfitting by reducing noises and random fluctuations during the training process. As a result, the DL model cannot overtrain all the samples and is forced to generalize to extract more information from the original dataset and achieve an accurate accuracy (Shorten and Khoshgoftaar, 2019). Duplication or oversampling is one form of data augmentation commonly used in DL; it allows adding more data by inflating the training dataset size. The data was split into 30% for validation and testing set each, and the remaining 40% was for the training set while considering the total classes of breast tissue (Figure 3B). The validation data can estimate the performance of the deep learning model with unseen data during the training process.

The biLSTM model accepts input as sequential or time-series data. However, the data used in this study is static in the form of a structured tabular array. Since the input has to become suitable to feed into the biLSTM model, a data augmentation procedure is applied by creating multiple slightly modified copies of each class in the dataset.

**Figure 4:**
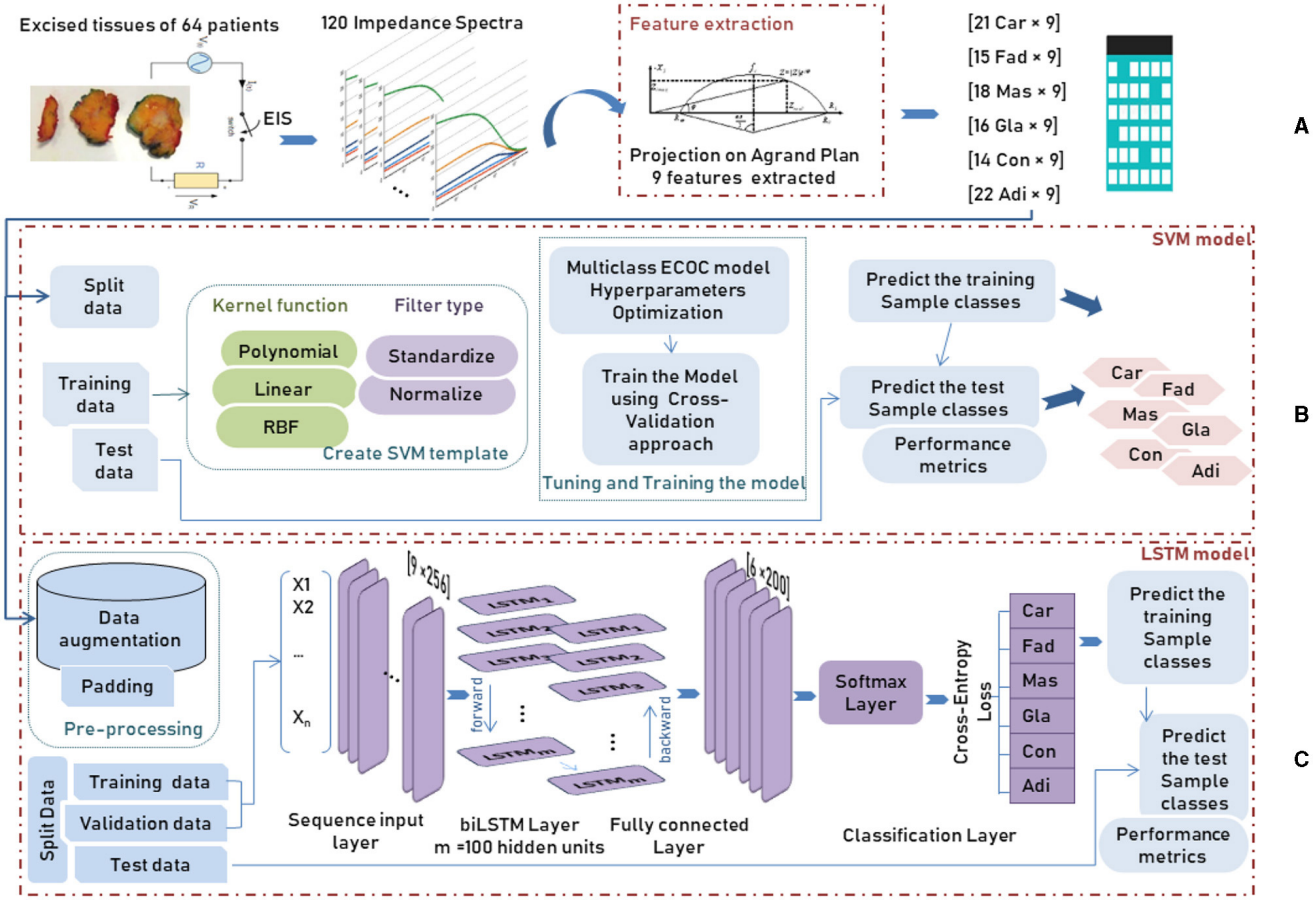
Workflow of an AI system for tissue classification using two different methods SVM and biLSTM, showing: **(A)** data preprocessing, **(B)** SVM method, and **(C)** biLSTM method.

After data augmentation, where a duplication operator was applied on each class of breast tissue, a padding or truncating step was applied to the data split into mini-batches with 256 samples long. The choice of small mini-batches size is important as it directly affects the training stability and the model performance. After data pre-processing, training and validation data were introduced to the LSTM model, consisting of 5 main components: the sequence input layer, bidirectional LSTM (biLSTM) layer, fully connected layer, softmax layer and classification layer, as shown in Figure 4C. First, the sequence input layer of size [256 × n], where *n* is the number of features, is passed through the biLSTM layer that learns bidirectional dependencies by interpreting the input data in both forward and backward directions between every step in order to map the input data into 100 features (hidden units). Next, the fully connected layer maps the output of the preceding layer by multiplying it by the weight matrix and adding a bias vector to obtain an output data size representing the six breast tissue classes. Next, the softmax layer interprets the input data with its activation function as a probability distribution followed by the cross-entropy loss function that computes the difference between these probabilities of each class in the classification layer.

### 2.4. Electrical impedance of breast tissue

Biological tissue can conduct electrical current with the association of impedance parameters. To understand these electrical properties more, it is important to remember some basics of tissue composition. Biological tissue comprises extracellular medium and cells; the latter consists of the cell membrane and intracellular medium composed of ionic solution and the extracellular medium. These ions in the solution are responsible for the cell's electrical conductivity (Hope and Iles, 2003). It was remarkable that the cell membrane acts dependently over the frequency in a simple RC circuit. Lower frequencies allow the cellular membrane to act like an insulator while the impedance is more resistive. At a higher frequency, the ability of the cell membrane to pass an electrical current is more important while the impedance decreases. In conclusion, biological tissues react dependently to the frequency after applying electrical current. Therefore, choosing the frequency range is important, especially for tumor detection.

## 3. Results and discussion

For the current study, two AI approaches are adopted to classify six classes of breast tissue to detect carcinoma breast cancer using impedance characterization data. The traditional machine learning algorithm is applied first, followed by the deep learning algorithm. SVM and LSTM models are evaluated using standard metrics: accuracy, sensitivity, and precision. These evaluation measures compare the true-positive rate and false-positive rate; they are commonly used to evaluate the performance of the system, mostly related to the identification of patterns (Powers, 2011). They are defined by the following equations (6–11):


(6) $$\begin{array}{l} Accuracy=\frac{TP+TN}{TP+TN+FP+FN} \end{array}$$

(7) $$\begin{array}{l} Sensitivity=\frac{TP}{TP+FN} \end{array}$$

(8) $$\begin{array}{l} Specificity=\frac{TN}{TN+FP} \end{array}$$

(9) $$\begin{array}{l} Precision=\frac{TP}{TP+FP} \end{array}$$

(10) $$\begin{array}{l} F\text{\_}measure=2\times \frac{Precision\times Sensitivity}{Precision+Sensitivity} \end{array}$$

(11) $$\begin{array}{l} G\text{\_}mean=\sqrt{Sensitivity\times Specificity} \end{array}$$

where TP, TN, FP and FN are true positive, true negative, false positive and false negative, respectively.

The first experiments were conducted with the ML-SVM and DL-LSTM models to assess the performance of the breast tissue classification system and, thus, breast cancer detection. The numerical results of the two initial experiments are reported in Table 3. As can be seen, the classification accuracy of the bi-LSTM model is 97.95%, which surpasses those of the CV-multiclass SVM model.

**Table 3:** Evaluation of two different AI model results using the whole impedance features.

**Models**	**Accuracy**	**Sensitivity**	**Specificity**	**Precision**	**F_measure**	**G_mean**
	**(%)**					
CV-multiclass SVM	76.19	100	70.59	44.44	61.54	84.02
Bidirectional LSTM	96.67	80.00	100	100	80.89	89.44

**Figure 5:**
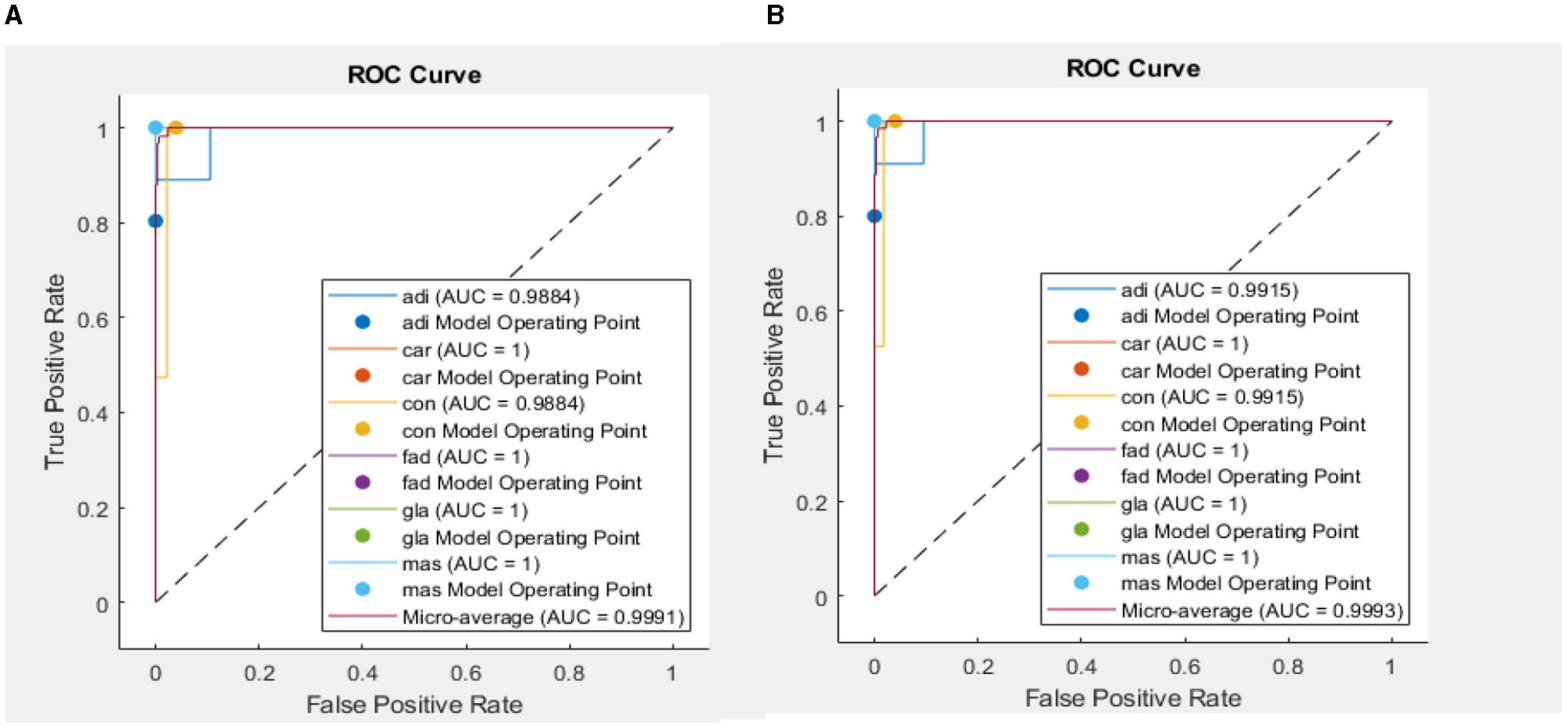
ROC (AUC) curve of DL model for test dataset **(A)** and training dataset **(B)**.

In addition, when the evaluation was performed over precision, specificity and all the remaining scores, it was seen that the bi-LSTM model yielded the highest scores among others. Table 4 compares the current literature results. Linear discriminant (Estrela da Silva et al., 2000) and backpropagation neural network BPNN (Helwan et al., 2017) models were evaluated using the same database adopted in this study. The results indicate that the proposed LSTM model achieved an improved recognition rate on testing with carcinoma discrimination of 100% compared to the other studies. Figure 5 represents the ROC (AUC) curve to evaluate the performance of LSTM model with the unknown data (b) and the training data (b), it is clearly shown that the model can perfectly predict all classes with more than 0.99 AUC value.

**Table 4:** Comparison of the proposed method with the reported results.

**Models**	**Recognition rate on testing (%)**	**Carcinoma discrimination (%)**
Linear discriminant classifier (Estrela da Silva et al., 2000)	~92	>86
BPNN1 (Helwan et al., 2017)	83.33	-
BPNN2 (Helwan et al., 2017)	83.33	-
BPNN3 (Helwan et al., 2017)	91.67	-
The proposed LSTM classifier	96.67	100

**Figure 6:**
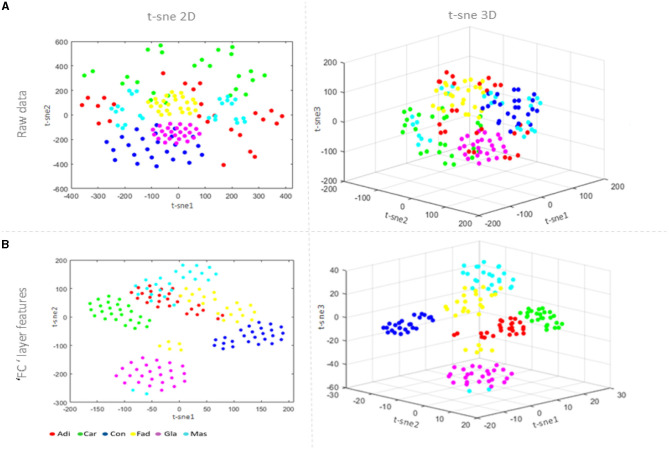
Feature visualization via 2D and 3D t-sne: **(A)** the raw data visualization; and **(B)** the fully connected layer visualization.

Visualizing the learning characteristics of LSTM model layers is important to understand the feature extraction process inside the model clearly. The idea is to reduce dimensionality using the t-distributing stochastic nearest neighbor embedding t-sne technique (van der Maaten and Hinton, 2008) by converting high-dimensional data into two or three-dimensional data that can be displayed in a scatterplot, as shown in Figure 6. A comparison is performed with the raw data visualization in Figure 6A and the fully connected layer feature visualization in Figure 6B. The regions that define breast tissue classes have gradually become closer, unlike those with different classes that become separable. From examining Figure 6A, it can be observed that the same samples of different classes are misclassified.

LSTM model was performed using different configurations to investigate every EIS feature separately. This step consisted of classifying the six breast tissue classes to explore the discriminating capability of EIS features. The proposed model demonstrates its reliability by achieving a 100% recognition rate among all classes for four different impedance features and 100% of carcinoma detection overall impedance features except for the area under Spectrum, as shown in Table 5.

**Table 5:** Evaluation of the LSTM model among each EIS feature.

**EIS features**	**Accuracy**	**Specificity**	**Precision**	**F_measure**	**Correct tissues identification**
		**(%)**			
P	98.29	97.95	90.70	95.12	Fad + Gla + Mas + Adi
HFS	16.67	20	20	86.1	-
DA	51.50	61.80	0	-	Car
AREA	83.33	80.00	50.00	66.67	Con + Fad + Gla + Mas
A/AD	98.42	98.16	91.32	95.47	Mas + Con + Adi + Car
IPmax	80.38	76.45	45.92	62.94	Mas + Con + Adi + Car
IO	100	100	100	100	Mas + Gla + Con + Adi + Fad + Car
PA500	16.67	20	0	0	-
DR	100	100	100	100	Mas + Gla + Con + Adi + Fad + Car

## 4. Conclusion

The results reported in this paper are a proof of concept of technology for non-invasive, early and rapid detection and differentiation of breast cancer based on Electrical Impedance characterization augmented with Deep Learning. The technique has the potential for early and rapid breast cancer detection due to the capability to characterize the evolution of the electric and dielectric properties of breast tissue from healthy to malignant, premalignant until cancerous tissue. Thanks to the progression of machine learning and deep learning techniques that help us to create a high-performance model to analyze EIS measurement. LSTM model has proved to be the best in breast tissue classification with ultimate accuracy; on top of that, I0 and DR impedance features carry the most relevant EIS characterization that can separate every single tissue of the breast and hence detect the cancerous one. In the future, more impedance spectroscopic data is required to investigate better the information contained in EIS mappings, under consideration of a specific logistic plan and clinical protocols. Moreover, data could be spread across multiple sessions of breast cancer stages, considering the diversity of patient gender, age, or medical conditions. Therefore, creating an accurate EIS biological model of breast cancer could be an effective tool for AI-based clinical decision-making.

## Data availability statement

The original contributions presented in the study are included in the article/Supplementary material, further inquiries can be directed to the corresponding author.

## Author contributions

SS and MM wrote the main manuscript text, created data visualization outputs, analyzed the results, and reviewed the manuscript. SA and AL reviewed and proofread the draft manuscripts. SS created the web scraping script, collected the data, conducted evaluation, and validation experiment(s). MM supervised the research with ZL as an AI expert advisor. All authors contributed to the article and approved the submitted version.
