# Supplementary material for: Early breast cancer detection and differentiation tool based on tissue impedance characteristics and machine learning
Source: Front Artif Intell. 2023 Sep 13;6:1248977. doi: 10.3389/frai.2023.1248977 (PMC10534039; doi:10.3389/frai.2023.1248977)
Supplement: Supplementary file 1 [file Data_Sheet_1.docx]

Supplementary Material

Early Breast Cancer Detection and Differentiation Tool Based on Tissue Impedance Characteristics and Machine Learning

Soumaya Ben Salem^1,2,^*, Samar Zahra Ali^2^, Anyik John Leo^2^, Zied Lachiri^1^, Martin Mkandawire^2^*

^1^SITI Laboratory, National School of Engineers of Tunis, University of Tunis El Manar, Tunis, Tunisia

^2^Department of Chemistry, School Science and Technology, Cape Breton University, Sydney, Nova Scotia, Canada B1P 6L2

*** Correspondence:** Soumaya Ben [Salem@cbu.ca](mailto:Salem@cbu.ca); [martin_mkandawire@cbu.ca](mailto:martin_mkandawire@cbu.ca)

# Supplementary Figures
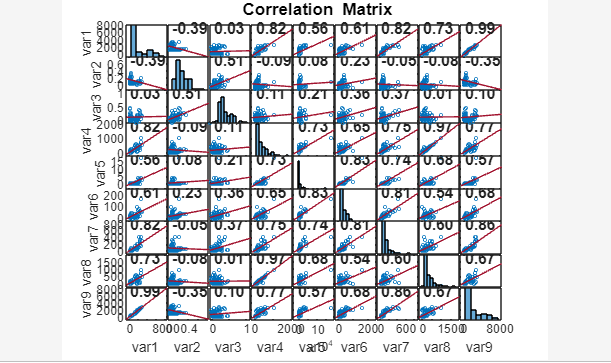


**Supplementary Figure 1.** The correlation matrix of the feature.


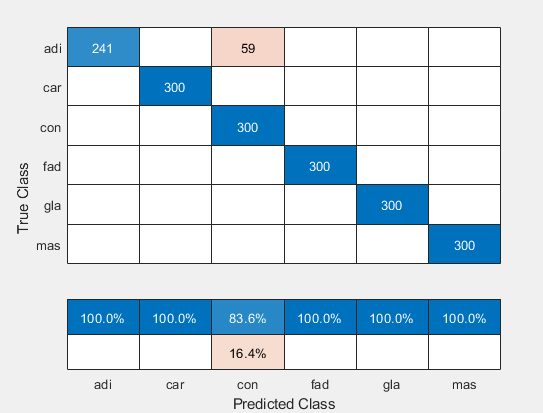


**Supplementary Figure 2.** The confusion matrix of the feature


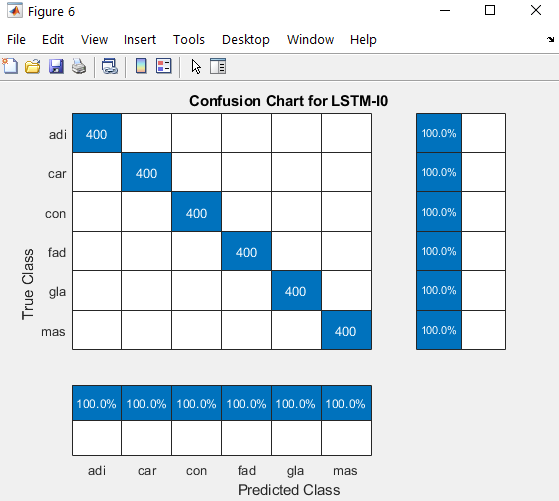


**Supplementary Figure 2.** The confusion matrix of the features I0 and DR. I0 and DR impedance features carry the most relevant EIS characterization that can separate every single tissue of the breast and hence detect the cancerous ones.
